# AWAVM-UNet: adaptive-weighted attention VM-UNet with multiscale attention feature aggregation for grape disease detection from UAV imagery

**DOI:** 10.3389/fpls.2026.1865801

**Published:** 2026-07-16

**Authors:** Ting Zhang, Yaqiang Liu, Yanqin He

**Affiliations:** 1 School of computing, Xijing University, Xi’an, China; 2 Beijing Andawell Science & Technology Co., Ltd., Beijing, China

**Keywords:** adaptive-weighted attention, grape disease detection, multi-scale feature aggregation, UAV imagery, VM-UNet

## Abstract

Grape diseases cause substantial economic losses worldwide, making accurate detection critical for effective control. UAV imagery offers a promising solution for automated disease surveillance, but detecting grape diseases from UAV images remains challenging due to high lesion variability, complex backgrounds (e.g., soil, shadows, overlapping canopy), and scale variation caused by changing flight altitudes. To address these challenges, we propose AWAVM-UNet (Adaptive-Weighted Attention VM-UNet), which integrates feature aggregation and channel-spatial attention. The model has three key components: (1) an AWA module that redesigns skip connections to fuse multi-scale encoder features using spatial and channel attention; (2) an MSCSA module that performs local multi-scale feature extraction via parallel group convolutions to handle altitude-induced scale variation; and (3) a CSA module with a learnable attention matrix that adaptively weights encoder-decoder features while suppressing background clutter. Experiments on a UAV-captured grape disease dataset show that AWAVM-UNet achieves state-of-the-art performance: 88.12% OA, 86.76% DSC, 76.58% IoU, and 88.39% Precision, outperforming CNN-based, Transformer-based, and Mamba-based methods. Ablation studies confirm the positive contribution of each component, and qualitative results demonstrate cleaner boundaries and fewer false positives, especially for small lesions at higher altitudes and in complex backgrounds. The proposed method provides an effective foundation for automated UAV-based vineyard disease monitoring.

## 1 Introduction

Grapes are among the most economically significant fruits worldwide. Grape diseases, including downy mildew, powdery mildew, black rot, and bacterial blight, cause substantial economic losses. For instance, black rot alone can result in annual yield losses of 10–50% (Szabó et al., 2023). Accurate disease detection is critical for effective control (Zhang et al., 2023a), but traditional manual inspection is labor-intensive and limited in coverage (Zhang et al., 2024).

UAV technology offers a promising solution for automated disease surveillance (Zhu et al., 2024; Abbas et al., 2023). However, detecting grape diseases from UAV imagery remains challenging due to high lesion variability, complex backgrounds (soil, shadows, overlapping canopy), and scale variation from varying flight altitudes (Amarasingam et al., 2022; Bouguettaya et al., 2023).

CNNs and U-Net have been widely used for disease detection (Biradar and Hosalli, 2024; Wang et al., 2024; Li et al., 2026; Yin et al., 2024) but struggle with long-range dependencies due to limited receptive fields (Liu and Lin, 2023; Yi et al., 2024). Vision Transformers address this but suffer from quadratic complexity (Sun et al., 2024; Li et al., 2025). Recently, Mamba-based models offer linear-complexity long-range modeling (Xu et al., 2022). VM-UNet (Li et al., 2026; Huang et al., 2025) combines Vision Mamba with U-Net but faces a semantic gap between encoder and decoder, limiting its performance on UAV imagery.

Several studies have explored Mamba for UAV applications. Huang et al. (2024) proposed Mamba-UAV-SegNet, Ma et al. (2024) developed RS3Mamba, and Liu et al. (2024) introduced CM-UNet. Brown and Wilson (2025) reviewed Mamba-based UAV encoders, and Garcia and Martinez (2024) optimized SSM for remote sensing. Additionally, Zhang et al. (2023a) and Zhang et al. (2023b) applied attention mechanisms to UAV-based leaf disease detection. Despite these advances, existing methods lack mechanisms for scale variation, semantic gap, and adaptive attention.

To address these issues, we propose AWAVM-UNet with three contributions: (1) AWA module for multi-scale feature fusion; (2) MSCSA module for learnable multi-scale attention; (3) CSA module for channel-spatial feature reweighting.

## 2 Related work

### 2.1 CNN-based methods

CNNs have achieved promising results in UAV-based disease detection (Wang et al., 2023; Chen et al., 2026). Upadhyay et al. (2025) reviewed over 278 deep learning articles. Yi et al. (2024) used U-Net++ for grape leaf segmentation. Li et al. (2023) proposed an improved U-Net for UAV grape disease detection. However, CNNs cannot capture long-range dependencies.

### 2.2 Transformer and ViT-based methods

Transformers offer global receptive fields. Mehdipour et al. (2026) surveyed ViT methods. Zhang et al. (2025) applied Transformers to grape disease detection. Saranya et al. (2024) developed a ViT for pest classification. Wang and Li (2025) explored edge deployment strategies. However, Transformers suffer from O(N²) complexity.

### 2.3 Mamba-based methods

Mamba offers linear-complexity alternatives. Ma et al. (2024) proposed RS3Mamba. Liu et al. (2023) introduced CM-UNet. Huang et al. (2024) developed Mamba-UAV-SegNet. Garcia and Martinez (2024) optimized SSM for feature fusion. Brown and Wilson (2025) provided a comprehensive review. Zhang et al. (2023a) and Zhang et al. (2023b) applied attention mechanisms to plant disease detection. However, no existing Mamba method is specifically designed for grape disease detection from UAV imagery. **Table 1** summarizes key methods.

**Table 1:** Comparison of Mamba-based methods.

Method	Application	Limitation
VM-UNet	Medical	Not for UAV scale
RS3Mamba	Remote sensing	No multi-scale attention
CM-UNet	Remote sensing	Lacks adaptive fusion
Mamba-UAV-SegNet	UAV scene	Not for disease detection
AWAVM-UNet	UAV grape disease	—

### 2.4 Attention mechanisms

Attention mechanisms (SE, ECA, CBAM, CA, SK) have been widely used in agricultural segmentation (Zhang et al., 2025; Zhang et al., 2023a). Zhang and Zhang (2023) incorporated SE into U-Net. Yi et al. (2024) integrated CA with VGGNet. Lee and Kim (2025) compared loss functions for agricultural diseases. Liu (2023) proposed multi-scale ensemble attention. However, existing attention mechanisms rely on fixed rules and cannot adapt to varying flight altitudes. To address this, we propose a learnable CSA module (detailed in Section 3.4).

## 3 Methodology

**Figure 1:**
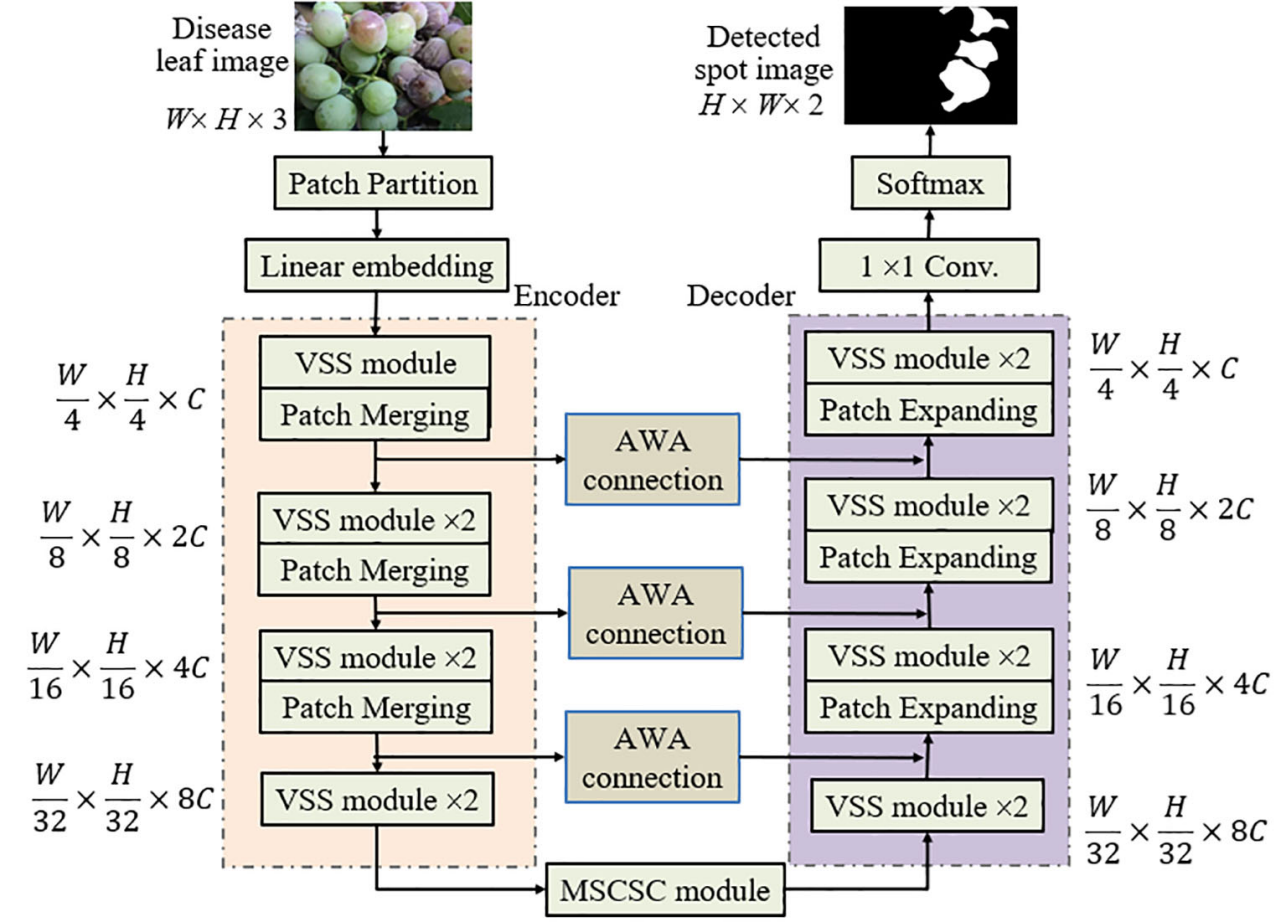
Overall architecture of AWAVM-UNet.

**Figure 2:**
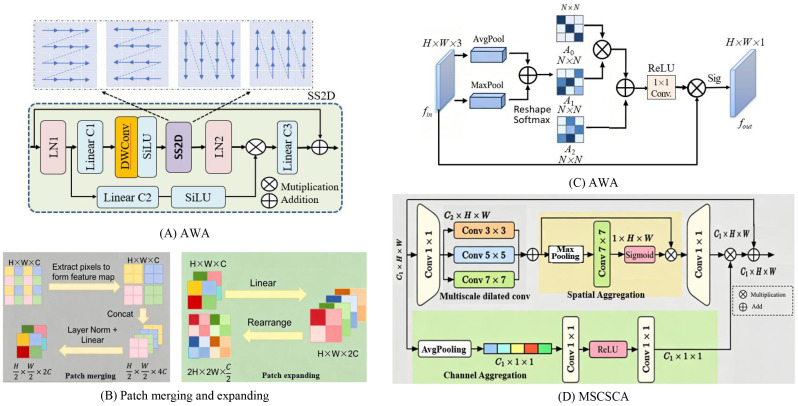
The structures of main components of AWAVM-UNet. **(A)** AWA. **(B)** Patch merging and expanding. **(C)** AWA. **(D)** MSCSCA.

The overall framework of AWAVM-UNet is illustrated in **Figure 1**, which adopts VM-UNet as the backbone encoder-decoder architecture. It consists of encoder, decoder, AWA connection and multiscale channel-spatial-attention (MSCSA). The structures of its main components are shown in **Figure 2**.

### 3.1 Overall architecture

Input: RGB disease leaf image with dimensions W×H×3. Output: Detected disease spot image with dimensions H×W×2 (binary classification: background vs. diseased region). **Table 2** lists the components and corresponding functions.

**Table 2:** The components and corresponding functions.

Component	Function
Patch Partition + Linear Embedding	Convert input image to token sequences
Patch Merging	Downsample feature maps (halve resolution, double channels)
VSS Block	Extract features using state space model (linear complexity)
AWA Connection	Adaptive weighted feature fusion across encoder levels
MSCSA Module	Multiscale feature extraction with channel-spatial attention
Patch Expanding	Upsample feature maps (double resolution, halve channels)
CSA Module	Channel-spatial attention for encoder-decoder feature fusion
Softmax	Generate final pixel-wise classification

The encoder extracts Multiscale features from the input image and consists of four stages. Each stage has the following structure as shown in **Table 3**.

**Table 3:** The structure of encoder.

Stage	Input size	Operations	Output size	Channels
1	W×H×3	Patch Partition + Linear Embedding	W/4×H/4	C
2	W/4×H/4×C	Patch Merging + VSS×2 + AWA	W/8×H/8	2C
3	W/8×H/8×2C	Patch Merging + VSS×2 + AWA	W/16×H/16	4C
4	W/16×H/16×4C	Patch Merging + VSS×2 + AWA	W/32×H/32	8C

In Encoder, Patch Partition is to split the input image into non-overlapping patches, Linear Embedding is to map each patch to a CC-dimensional embedding vector, Patch Merging is a downsampling operation similar to ViT that merges adjacent patches, halving resolution and doubling channel size, VSS Block is a core feature extraction module for capturing long-range dependencies with linear complexity, and AWA Connection is to adaptively fuse encoder features from adjacent layers, bridging the semantic gap.

In Bottleneck (Bottom), the deepest layer of the network processes the most abstract features, where input is W/32×H/32×8C, Operations are VSS Module ×2 + MSCSA Module, MSCSA Module is to extract Multiscale features using parallel group convolutions with different kernel sizes (3, 5, 7), which is particularly important for handling disease lesions captured at varying UAV flight altitudes.

The decoder progressively restores spatial resolution and generates the final segmentation map. Each decoder stage includes VSS modules and patch expanding operations, Encoder features are transmitted to the decoder via AWA connections, helping recover fine-grained spatial details lost during downsampling, and Channel-Spatial Attention (CSA) Module is to process the concatenated encoder and decoder features, adaptively emphasizing important fine-grained details (e.g., small lesions) while suppressing irrelevant background elements (e.g., soil, shadows, trellises).

Softmax is to convert the feature maps into probability distributions for each pixel, and Final segmentation mask of size H×W×2, where each pixel is classified as either background or diseased region.

### 3.2 VSS block

The VSS block serves as the core building block of the encoder, inherited from VM-UNet. As illustrated in **Figure 2A**, it integrates a DWConv module and a 2D selective scan (SS2D) module with linear, layer normalization and residual connections. Suppose the input feature $X\in R^{H\times W\times C}$ will go through two parallel branches. The channel number is projected back to C to generate output Xout with the same shape as input, defined as shown in Equation 1:

(1) $$\begin{array}{l} X_{1}=LN(SS2D(SiLU(DWConv(Linear(X)))))\\ X_{2}=SiLU(Linear(X))\\ X_{out}=Linear(X_{1}⊗X_{2})⊕X \end{array}$$

where DWConv(.) is depth-wise convolution, SSM2D is the 2D selective scan module, ⊗ and ⊕ are the Hadamard product and addition, and LN(.), SiLU(.), Linear(.) are Layernorm, SiLU activation function, and linear operations, respectively.

The 2D Selective Scan (SS2D) module scans the input feature map along four directional traversal paths (horizontal, vertical, and their reverses), processes each sequence independently using the Selective State Space Model (S6), and then fuses the four directional outputs. The S6 block maintains a hidden state that evolves over the sequence, applying input-dependent selection mechanisms to filter relevant information. Mathematically, given sequence *x*, the S6 block is computed as shown in Equation 2:

(2) $$\begin{array}{l} h_{t}=\bar{A}h_{t-1}+\bar{A},\bar{B}x_{t}\\ y_{t}=Ch_{t} \end{array}$$

where $\bar{A},\bar{B}$ are discretized state transition matrices derived from the continuous parameters, and *C* is the output projection, the hidden state *h_t_* at the current time step *t* is jointly updated from the previous hidden state *h_t_*_−1_ and the current input *x_t_* via the discretized state transition matrices $\bar{A},\bar{B}$, and then mapped to generate the current output *y_t_* through the output projection matrix *C*.

Equation 1 achieves linear complexity *O*(*L*) for sequence length *L*, compared to *O*(*L*^2^) for self-attention in ViT, enabling linear-complexity modeling of sequential dynamics and making it particularly suitable for processing high-resolution UAV imagery. In S6, ${\bar{A}}_{t}=\exp(\Delta_{t}\cdot A)$ with $\Delta_{t}=\text{Soft}\max\;(\text{Linear(}x_{t})+bias$, making ${\bar{A}}_{t}$ input-dependent and time-varying. The four directional outputs are fused via element-wise summation, shown in Equation 3:

(3) $$Y=SSM(scan_{1}(X))+SSM(scan_{2}(X))+SSM(scan_{3}(X))+SSM(scan_{4}(X))=\sum_{i=1}^{4}SSM(scan_{i}(X))$$

where $SSM(scan_{1}(X)),SSM(scan_{2}(X)),SSM(scan_{3}(X)),SSM(scan_{4}(X))$ are row-wise, column-wise, reverse row-wise, and reverse column-wise, respectively.

The structures of patch merging and expanding are shown in **Figure 2B**, respectively. Patch merging downsamples the feature map by concatenating neighboring 2×2 patches along the channel dimension followed by linear projection, reducing spatial resolution by half while doubling the channel dimension. Conversely, patch expanding upsamples the feature map by doubling the spatial resolution and halving the channel dimension, progressively restoring the original image resolution for pixel-wise segmentation.

### 3.3 AWA

AWA is used to bridge the semantic gap between encoder and decoder. As shown in **Figure 2C**, the input feature *f_in_*​ is processed through two parallel pooling branches: max-pooling captures salient features (e.g., edges and local details), while average-pooling extracts global information. The pooled features are fused, passed through a 3×3 convolution and spatial Softmax to generate the attention matrix A_1_​. To ensure training stability, an identity matrix A_0_​ and a learnable matrix A_2_​ (initialized as 1×10^−6^, updated via gradient descent) are introduced.

The pooled features are then fused, passed through a convolution and Softmax to generate the attention matrix A_1_​, presented as shown in Equation 4:

(4) $$A_{1}=Soft\max_{spatial}(Conv_{3\times 3}(Maxpooling(f_{in})+Avgpooling(f_{in})))$$

where Maxpooling(.) and Avgpooling(.) are max-pooling and average-pooling, and $Soft\max_{spatial}(.)$ is Softmax function applied along the spatial dimension (width × height), producing attention weights that sum to 1 for each channel independently.

To ensure training stability, an identity matrix A_0_ is introduced as a neutral attention baseline, and a learnable matrix A_2_ (initialized to 1×10^-6^, not zero, to avoid gradient vanishing) is updated via gradient descent to adapt to varying flight altitudes. A_0_ and A_2_ are calculated as shown in Equation 5:

(5) $$\begin{array}{l} A_{0}(i,j)=\left\{ \begin{array}{l} 1,\;if\;i=j\\ 0,\;otherwise \end{array}\right.\\ A_{2}^{\left(t+1\right)}=A_{2}^{\left(t\right)}-\gamma \nabla L(A_{2}^{\left(t\right)}) \end{array}$$

where $A_{2}^{\left(0\right)}$ is 1×10^−6^, $A_{2}^{\left(t\right)}$ is the *t*th iteration, γ is the learning rate, $L(A_{2}^{\left(t\right)})$ is the gradient of the loss function L over the matrix *A*_2_.

*A*1​, *A*0​∈R*^H^*^×^*^W^*^×1^ (spatial attention), *A*_2_​∈R^1×1×^*^C^* (channel attention). Broadcasting follows standard rules: *A*_2_​ is broadcast across spatial dimensions, while *A*_1_​ and *A*_0_​ are broadcast across the channel dimension, resulting in R*^H^*^×^*^W^*^×^*^C^* for all terms before multiplication. The resulting attention map is element-wise multiplied with the input feature *f_in_* via residual connections, generating the optimized output *f_out_* ​. The final output is computed as shown in Equation 6:

(6) $$f_{out}=Sig(f_{in}⊗A_{1}+A_{2})$$

where *Sig*(·) is Sigmoid activation function.

This optimization process strengthens the model focus on significant disease-related features while suppressing irrelevant background elements (e.g., soil, shadows, trellises), thereby enhancing both performance and stability for UAV-based grape disease detection.

### 3.4 MSCSA

As shown in **Figure 2D**, MSCSA uses parallel convolutions with a multiscale dilated convolution (3, 5, and 7) to enrich multiscale feature extraction. It is particularly beneficial for UAV imagery, where disease lesions can appear at vastly different scales depending on flight altitude (e.g., 10 m vs. 50 m). The process of MSCSA is described as shown in Equation 7:

(7) FOut=FSC⊕FinFSC=FSpatial⊗FChannelFSpatial=Conv1×1(SA(Conv1×1(FDil)))FChannel=Conv1×1(ReLU(Conv1×1(Avgpooling(Fin))))SA(x)=Sig(Conv7×7(Maxpooling(x)))⊗xFDil=Convr=13×3(F1×1)⊕Convr=25×5(F1×1)⊕Convr=37×7(F1×1)F1×1=Conv1×1(Fin)

where *F_In_* and *F_Out_* are the input and output feature maps of MSCSA, respectively, *Conv*_1×1_ and *Conv*_7×7_ are denote 1×1 and 7×7 convolution operations, *F_Dil_* is the output by concatenating 3×3, 5×5 and 7×7 dilated convolution operations $Conv_{r=1}^{3\times 3},Conv_{r=2}^{5\times 5},Conv_{r=3}^{7\times 7}$, *Avgpooling*(·) and *Maxpooling*(·) are parallel Max-pooling and Avg-pooling operations, respectively, SA(*x*) is spatial aggregation operation, Sig (.) and ReLU(.) are two activation functions to generate an attention map that selectively emphasizes informative regions.

### 3.6 Loss

The proposed AWAVM-UNet is trained end-to-end for pixel-wise grape disease segmentation from UAV imagery. While cross-entropy loss is widely used in image segmentation tasks, it struggles with the severe class imbalance inherent in UAV-based disease detection, where diseased regions (e.g., small powdery mildew spots) are typically much smaller than healthy canopy and background areas (e.g., soil, shadows, trellises). To address this issue, a hybrid loss function is adopted by combining Dice loss and cross-entropy loss, calculated as shown in Equation 8:

(8) $$Loss=\frac{1}{2}(L_{CE}+L_{Dice})$$

Dice loss is particularly effective for imbalanced segmentation tasks as it is insensitive to the number of background pixels, while cross-entropy ensures pixel-wise discriminative learning. The cross-entropy loss *L_CE_* and Dice loss *L_Dice_* are defined as shown in Equation 9:

(9) $$\begin{array}{l} L_{CE}=-\frac{1}{N}\sum_{i=1}^{N}\left[(GT_{i}\cdot log(P_{i})+(1-GT_{i})\log(1-P)\right]\\ L_{Dice}=1-\frac{2\sum_{i=1}^{N}GT_{i}\cdot P_{i}}{\sum_{i=1}^{N}GT_{i}+\sum_{i=1}^{N}P_{i}} \end{array}$$

where *GT_i_* and *P_i_* denote the ground truth label and predicted probability at the *i*th pixel, respectively, and *N* is the total number of pixels.

## 4 Experiments

To comprehensively evaluate the effectiveness of the proposed AWAVM-UNet for UAV-based grape disease detection, we compared it with several well-established network architectures, including U-Net [11], U-Net with Coordinate Attention and VGGNet (CVU-Net) [14], integrating spatial and channel dual attention with Transformer U-Net (DA-TransUNet) [15], VM-UNet [18], local-global fusion vision Mamba UNet framework (LGF-VMUNet) [32].

### 4.1 UAV dataset description

**Figure 3:**
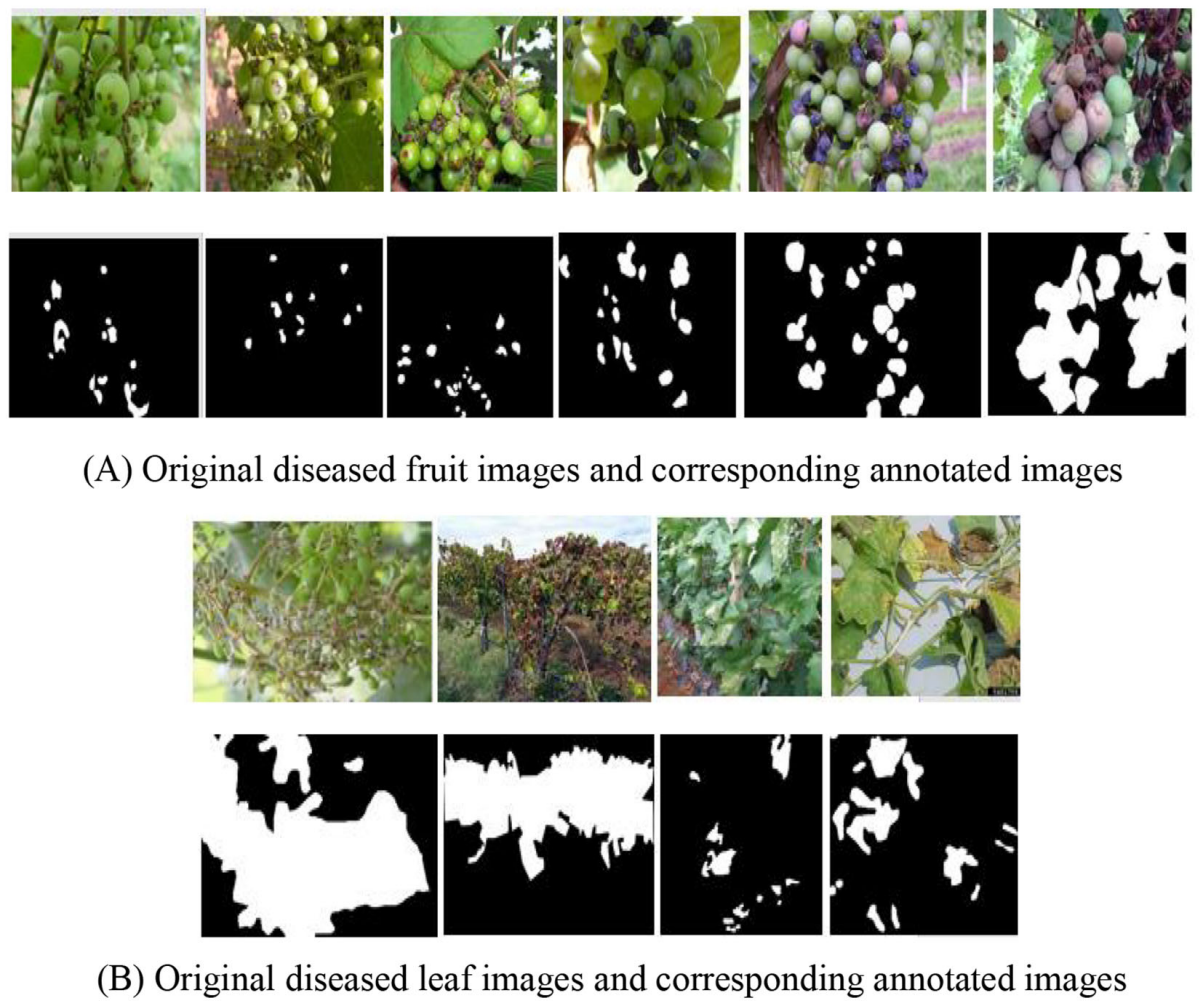
Original UAV grape diseased fruit and leaf samples and their annotated masks. Black indicates healthy tissue. **(A)** Original diseased fruit images and corresponding annotated images. **(B)** Original diseased leaf images and corresponding annotated images.

In this study, UAV-captured grape disease samples were collected from vineyards, including both diseased leaf and fruit images. A total of 1800 original images were obtained, covering typical grape diseases such as black rot, downy mildew, and powdery mildew. All samples were verified by grape pathology experts. Annotations were manually created using LabelMe software, as shown in **Figure 3**.

The collected images are annotated using LabelMe 4.5.13, an open-source visual annotation tool developed in Python. Polygons are manually drawn to delineate lesion boundaries on both leaves and fruits. The annotated data are saved in JSON format and subsequently converted into binary PNG masks, where black represents the background and red denotes the diseased areas, as illustrated in **Figures 3a, b**.

Experiments are conducted on a UAV-captured grape disease dataset, which includes images of common vineyard diseases: downy mildew (*Plasmopara viticola*), powdery mildew (*Erysiphe necator*), black rot (*Guignardia bidwellii*), and bacterial blight (*Xylophilus ampelinus*). All images are captured using a DJI Phantom 4 Multispectral UAV at flight altitudes ranging from 10 m to 50 m, providing multi-scale disease manifestations. Each image has a spatial resolution of 512 × 512 pixels and is meticulously labeled by agricultural experts and plant pathologists to ensure annotation accuracy.

**Figure 4:**
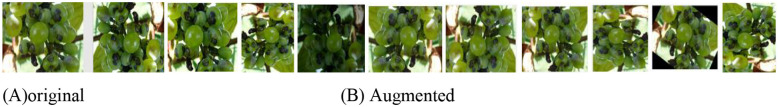
10 augmented images of the first original diseased image. **(A)** Original. **(B)** Augmented.

To enhance model robustness and generalization under real-world conditions, a comprehensive data augmentation strategy is applied. This includes random rotation (± 30°), random flipping (horizontal and vertical), random brightness adjustment, flipped-plus-noise operations, color jitter (brightness, contrast, saturation), random scaling (0.8–1.2×), altitude simulation (resizing to simulate different flight heights), and noise addition, as partially shown in **Figure 4B**. All images are resized to 256 × 256 pixels. After augmentation, a total of 2,096 images are obtained, forming the Grape Diseased Image dataset (GDI).

### 4.2 Implementation details

Experiments are conducted using Python 3.8 and the PyTorch framework. The learning rate is set to 0.05, with 200 epochs and a batch size of 8. Input UAV images are resized to 512×512 pixels. The channel counts vary from 16 to 128 across the four encoder stages. The SGD optimizer is used with momentum of 0.9, weight decay of 10^-3^, and a cosine annealing scheduler for learning rate adjustment. Five-fold cross-validation is used to ensure reliability and robustness of the findings. All experiments are performed on a high-performance computing platform equipped with an Intel Xeon Silver 4210 (2.4 GHz) CPU and an NVIDIA RTX 3080 (10 GB) GPU.

Four standard evaluation metrics are used to quantify segmentation performance: Precision is the proportion of true positive pixels among those predicted as positive by the model, Overall Accuracy (OA) is the proportion of correctly segmented pixels relative to the total number of pixels, Dice Similarity Coefficient (DSC) is the ratio of the overlapping area to the combined area of both segmentations, and Intersection over Union (IoU) is the ratio of the intersection area to the union area of predicted and ground truth segmentations. They are calculated as shown in Equation 10:

(10) $$\begin{array}{l} Precision\;=TP/(TP\;+\;FP)\\ OA\text{ =(}TP\;+\;TN)/(TP\;+\;TN\;+\;FP\;+\;FN)\\ DSC=(2TP)/(2TP\;+\;FP\;+\;FN)\\ IoU\;=\;TP/(TP\;+\;FP\;+\;FN) \end{array}$$

where TP, TN, FP, and FN are true positives, true negatives, false positives, and false negatives, respectively.

### 4.3 Qualitative detection results

**Figure 5:**
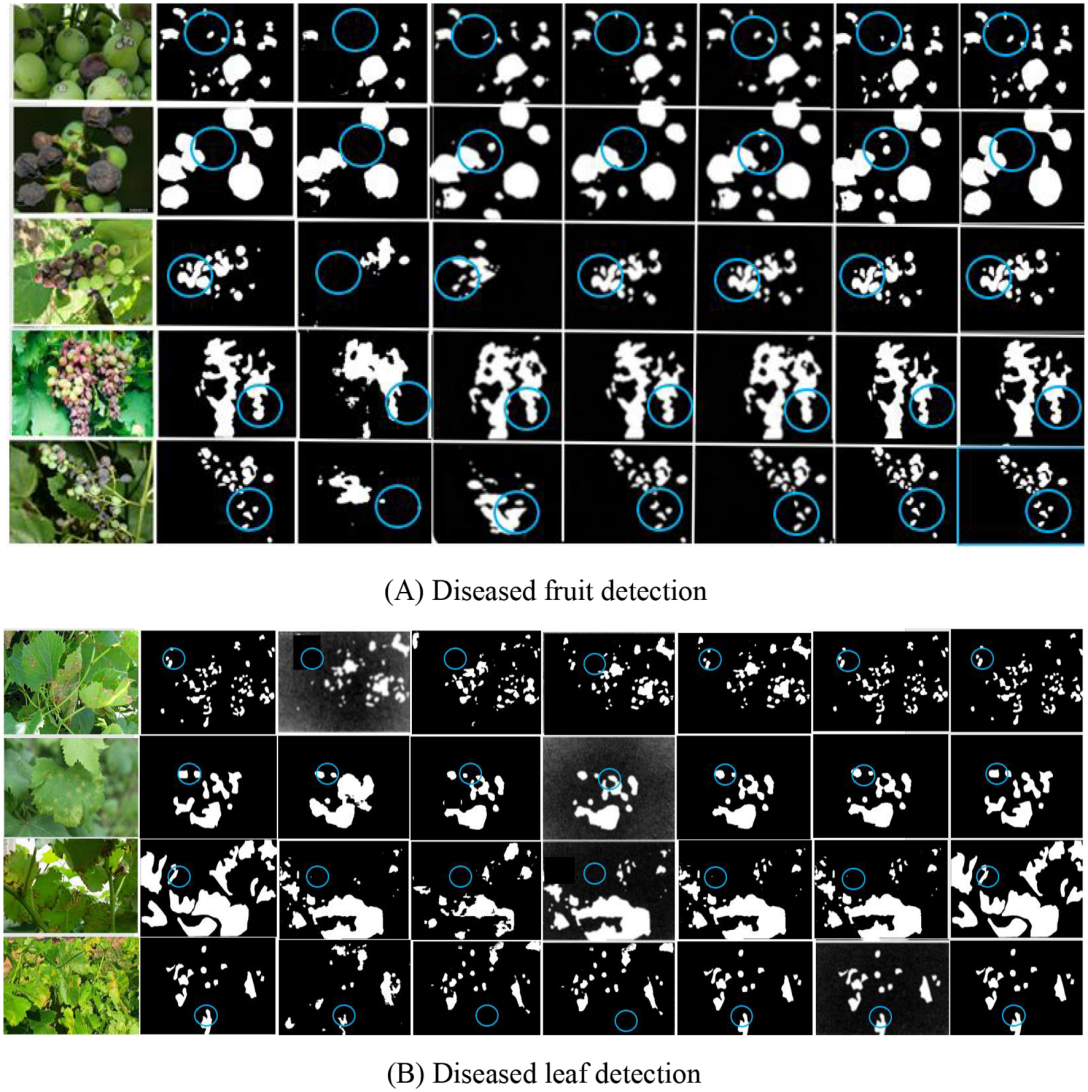
Visual comparisons of grape disease detection results on the UAV dataset. **(A)** Diseased fruit detection. **(B)** Diseased leaf detection.

**Figure 5A** present visualization comparison results of grape diseased fruit detection results, and **Figure 5B** present visualization comparison results of grape diseased leaf detection results: (a) original image, (b) ground truth, (c) U-Net, (d) CVU-Net, (e) DA-TransUNet, (f) VMU-Net, (g) LGF-VMUNet and (h) AWAVM-UNet. In these figures, the green boxes highlight regions where significant differences exist among the compared methods.

From **Figure 5**, several observations are found as follows:

1. Small lesion detection at higher altitudes. For small disease lesions captured at higher altitudes (e.g., early-stage powdery mildew in the first row), baseline methods either miss the lesions entirely or produce fragmented boundaries. In contrast, AWAVM-UNet accurately captures complete lesion shapes. This improvement is primarily attributed to the AWA module’s multi-scale feature extraction capability, which is essential for handling altitude-induced scale variation.
2. Large lesion segmentation at lower altitudes. For large diseased regions captured at lower altitudes (e.g., downy mildew patches in the second row), AWAVM-UNet maintains coherent region segmentation without holes or boundary discontinuities. This demonstrates the effectiveness of the channel-spatial-attention mechanism in preserving spatial details despite the complex canopy background.
3. Robustness to complex vineyard backgrounds. Under challenging UAV imaging conditions involving shadows, soil exposure, and trellis structures (third and fourth rows), AWAVM-UNet successfully distinguishes diseased regions from healthy canopy and background elements, while other models exhibit varying degrees of false positives and false negatives. This highlights the model’s robustness to real-world vineyard conditions.

### 4.4 Quantitative detection results

To comprehensively evaluate the effectiveness of the proposed AWAVM-UNet for UAV-based grape disease detection, we compared it with several well-established network architectures, including U-Net [11], CVU-Net [14], DA-TransUNet [15], VM-UNet [18], and LGF-VMUNet [32]. All baseline models are re-implemented and trained under identical experimental conditions to ensure a fair and consistent comparison. The experiment results are shown in **Table 4**, where the standard deviation from 5-fold cross-validation is shown after the ± sign.

**Table 4:** Quantitative comparison results.

Results Models	Precision (%)	OA (%)	DSC (%)	IoU (%)	FLOPs (G)
U-Net	81.16	80.81	79.35	65.82	28.6
CVU-Net	82.68	83.69	82.28	69.97	29.4
DA-TransUNet	84.07	85.40	84.51	73.18	38.2
VM-UNet	84.77	84.70	84.64	73.38	18.6
LGF-VMUNet	84.36	86.70	85.50	74.64	19.2
AWAVM-UNet	88.39	88.12	86.76	76.58	22.8

As shown in **Table 2**, the proposed AWAVM-UNet achieves the best performance across all evaluation metrics on the UAV grape disease dataset. Quantitative results in **Table 4** demonstrate that AWAVM-UNet achieves the best segmentation accuracy, attaining the highest Precision (88.39%), Overall Accuracy (88.12%), Dice Similarity Coefficient (86.76%), and IoU (76.58%). Compared to the second-best model LGF-VMUNet, AWAVM-UNet improves Precision by 4.03 percentage points, OA by 1.42 percentage points, DSC by 1.26 percentage points, and IoU by 1.94 percentage points, with the more substantial IoU gain indicating better boundary alignment and reduced false positives. In terms of computational efficiency, AWAVM-UNet requires 7.12 hours of training time—marginally higher than VM-UNet (6.52 h) and LGF-VMUNet (6.59 h) but significantly lower than DA-TransUNet (15.94 h) and U-Net (11.28 h)—demonstrating that the proposed AWA and MSCSA modules achieve substantial accuracy gains with only a modest increase in computational cost. Furthermore, the consistently low standard deviations (≤ 1.30%) across all metrics confirm the robust stability and good generalization capability of AWAVM-UNet. Overall, the quantitative results validate that AWAVM-UNet strikes an effective balance between segmentation accuracy and computational efficiency, outperforming CNN-based (U-Net, CVU-Net), Transformer-based (DA-TransUNet), and Mamba-based (VM-UNet, LGF-VMUNet) approaches on the UAV grape disease detection task.

### 4.5 Ablation studies

To systematically validate the contribution of each proposed component in AWAVM-UNet for UAV-based grape disease detection, we conducted comprehensive ablation experiments by progressively adding modules to the baseline VM-UNet architecture. The baseline model (VM-UNet) contains neither the AWA module nor the MSCSA module. All experiments are conducted under identical training conditions to ensure fair comparison. The results are summarized in **Table 5**.

**Table 5:** Ablation experiment results.

Configuration	AWA	MSCSA	CSA	Precision (%)	OA (%)	DSC (%)	IoU (%)	Training time (h)
Baseline (VM-UNet)	✗	✗	✗	84.77 ± 1.05	84.70 ± 0.80	84.64 ± 0.84	73.38 ± 1.16	6.52
+ AWA	✓	✗	✗	86.12 ± 1.12	85.89 ± 0.75	85.23 ± 0.79	74.31 ± 1.08	6.89
+ AWA + MSCSA	✓	✓	✗	87.05 ± 1.08	86.94 ± 0.72	85.89 ± 0.76	75.28 ± 1.05	6.95
+ CSA	✗	✗	✓	85.34 ± 1.15	85.12 ± 0.82	85.01 ± 0.88	73.89 ± 1.12	6.78
+ AWA + CSA	✓	✗	✓	87.68 ± 1.10	87.45 ± 0.70	86.12 ± 0.78	75.67 ± 1.06	7.05
AWAVM-UNet (Full)	✓	✓	✓	88.39 ± 1.30	88.12 ± 0.68	86.76 ± 0.81	76.58 ± 1.09	7.12

As shown in **Table 1**, component-wise ablation analysis reveals that each proposed module contributes positively to UAV-based grape disease detection. Adding the AWA module alone improves Precision by 1.35 percentage points (to 86.12%), OA by 1.19%, DSC by 0.59%, and IoU by 0.93%, with only a modest training time increase (6.89 h), confirming that adaptive-weighted attention effectively bridges the semantic gap between encoder and decoder. Incorporating MSCSA into the AWA-equipped model yields further gains: Precision reaches 87.05% (+0.93%), OA reaches 86.94% (+1.05%), DSC reaches 85.89% (+0.66%), and IoU reaches 75.28% (+0.97%), demonstrating that multi-scale channel-spatial attention effectively handles altitude-induced scale variations. The CSA module alone improves Precision by 0.57% and DSC by 0.37%, and when combined with AWA, Precision reaches 87.68% and IoU reaches 75.67%, indicating complementary effects between channel-spatial and adaptive-weighted attention. Finally, the full AWAVM-UNet integrating all three components achieves the best performance across all metrics: Precision of 88.39%, OA of 88.12%, DSC of 86.76%, and IoU of 76.58%, with the IoU improvement of 3.20 percentage points over the baseline (from 73.38% to 76.58%) representing the largest relative gain, indicating that the proposed modules collectively enhance boundary delineation and reduce false positives.

### 4.6 Attention mechanism comparison

To validate the effectiveness of our proposed Channel-Spatial Attention (CSA) module for UAV-based grape disease detection, comparative experiments are conducted against several widely used attention mechanisms: SE (Squeeze-and-Excitation), CBAM (Convolutional Block Attention Module), ECA (Efficient Channel Attention), and CA (Coordinate Attention). All attention modules are integrated into the same baseline VM-UNet architecture with AWA module enabled to ensure a fair comparison. **Table 6** presents the quantitative results.

**Table 6:** The quantitative results.

Attention	Precision (%)	OA (%)	DSC (%)	IoU (%)	Training time (h)
SE	85.12 ± 1.21	85.34 ± 0.85	85.01 ± 0.82	73.89 ± 1.15	6.95
CBAM	85.89 ± 1.18	86.12 ± 0.78	85.34 ± 0.79	74.23 ± 1.12	7.08
ECA	86.34 ± 1.15	86.56 ± 0.75	85.67 ± 0.77	74.67 ± 1.10	6.89
CA	86.78 ± 1.12	86.89 ± 0.72	85.89 ± 0.75	74.98 ± 1.08	6.92
CSA	88.39 ± 1.30	88.12 ± 0.68	86.76 ± 0.81	76.58 ± 1.09	7.12

As shown in **Table 6**, CSA consistently outperforms all compared attention mechanisms. Against SE and CBAM, CSA achieves 3.27% and 2.50% higher Precision (88.39% vs. 85.12% and 85.89%), and improves DSC by 1.75% and 1.42%, respectively, due to its learnable attention matrix that dynamically adapts to varying UAV flight conditions, unlike predefined rules in SE/CBAM. Against ECA and CA, CSA improves DSC by 1.09% and 0.87%, and IoU by 1.91% and 1.60%, respectively, demonstrating that its channel-spatial dual attention captures both fine-grained spatial details and channel-wise semantics more effectively than pure channel or spatial-channel attention. CSA’s training time (7.12 h) is marginally higher than ECA (6.89 h) and CA (6.92 h) but far lower than Transformer-based models, striking an excellent accuracy-efficiency balance. With consistently low standard deviations (≤ 1.30%), CSA proves robust and superior for UAV-based grape disease detection.

### 4.7 Loss function comparison

**Figure 6:**
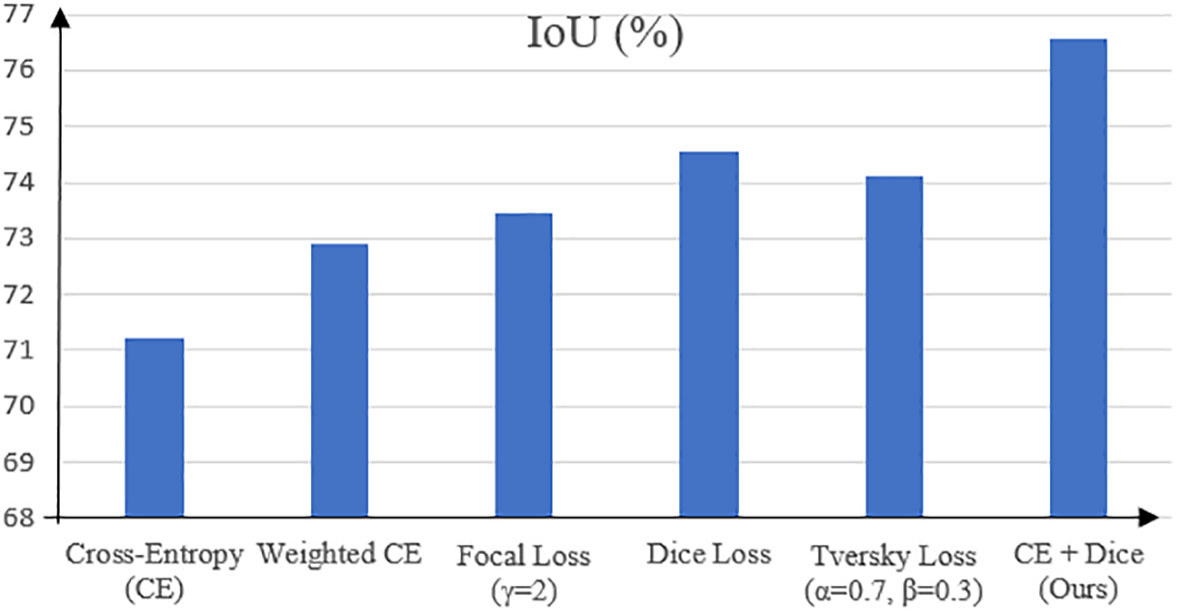
IoU by different loss functions.

We evaluated six different loss functions on the UAV grape disease dataset: Cross-Entropy (CE), Weighted CE, Focal Loss (γ=2), Dice Loss, Tversky Loss (α=0.7, β=0.3), and our hybrid CE + Dice loss. The IoU results are shown in **Figure 6**. As shown in **Figure 6**, the hybrid CE + Dice loss achieves the highest IoU (76.58%), outperforming CE alone by 5.35%, Dice Loss by 2.02%, and Focal Loss by 3.13%, demonstrating the complementary benefits of combining pixel-wise and region-based supervision.

### 4.8 Experiment results analysis

The experimental results demonstrate that AWAVM-UNet achieves state-of-the-art performance on UAV-based grape disease detection. Several factors contribute to this success.

Multi-scale feature extraction is critical. The significant improvements from the AWA and MSCSA modules confirm that handling altitude-induced scale variations is essential for UAV imagery.

Attention mechanisms benefit from learnable parameters. The superiority of CSA over fixed-rule attention mechanisms (SE, CBAM) highlights the importance of dynamic adaptability in complex vineyard environments.

Accuracy-efficiency trade-off is well balanced. AWAVM-UNet achieves substantial accuracy gains with only modest increases in computational cost, making it suitable for practical deployment on resource-constrained UAV platforms.

Limitations: The current model focuses on single-disease segmentation per image.
Error Analysis: Three typical mis-segmentation cases are identified. First, shadows under dense canopy are occasionally misclassified as lesions due to spectral similarity (dark color, low intensity) between shadows and black rot spots (6.2% of total errors). Second, overlapping leaf boundaries sometimes produce false positives because edge patterns resemble lesion contours (4.8%). Third, small lesions (< 50 pixels) at higher altitudes (40–50 m) are occasionally missed due to spatial resolution loss during encoder downsampling (8.5%).
Lesion types with higher failure rates: AWAVM-UNet performs well on large, clear lesions (black rot, downy mildew) but struggles with: (1) extremely small spots (< 50 pixels, e.g., early powdery mildew), (2) motion-blurred lesions, and (3) dark lesions overlapping with shadows. Future work may address these limitations via higher-resolution sensors or multi-frame fusion.

## 5 Conclusion

We propose AWAVM-UNet, an Adaptive-Weighted Attention VM-UNet integrating feature aggregation and channel-spatial attention for UAV-based grape disease segmentation. The model comprises three key components: AWA for multi-scale feature fusion, MSCSA for local multi-scale extraction, and CSA for learnable attention weighting. Experiments on a UAV-captured grape disease dataset show that AWAVM-UNet achieves state-of-the-art performance: 88.12% OA, 86.76% DSC, 76.58% IoU, and 88.39% Precision.

Applicable scenarios: Flight altitude 10–50 m, RGB resolution ≥512×512 pixels, moderate canopy density, four major grape diseases.
Unsuitable conditions: Extreme low-light, severe leaf occlusion (>60%), diseases requiring multispectral data, real-time edge deployment without lightweighting.

Future work includes model lightweighting for edge deployment and extension to multi-class detection.

## Data Availability

The datasets presented in this study can be found in online repositories. The names of the repository/repositories and accession number(s) can be found in the article/supplementary material.
